# Vital Statistics and Sanitation; Sewers and Sewage

**Published:** 1899-11

**Authors:** 


### TEXAS MEDICAL JOURNAL.

AUSTIN, TEXAS.

A MONTHLY JOURNAL OF MEDICINE AND SURGERY.

F. E. DANIEL, M. D., Editor.

Published Monthly at Austin, Texas, by Drs. Daniel and Hudson. Subscription price $1.00 a year in advance.

Eastern Representative: John Guy Monihan, St. Paul Building, 220 Broadway, New York City.

Official organ of the West Texas Medical Association, the Houston District Medical Association, the Austin District Medical Society, the Brazos Valley Medical Association, the Galveston County Medical Society, and several others.

### EDITORIAL.

VITAL STATISTICS AND SANITATION; SEWERS AND SEWAGE.—We devote considerable space in this issue to a reproduction of the circular from the Census Bureau outlining a plan for a "uniform system of mortality reports" because of the importance of the subject.

**AS TO STATISTICS AND SANITATION.**

Every physician who is interested in the great question of sanitation and sanitary reform,—and every one should be,—will read this circular carefully, and lend his aid and co-operation, however little it may be, to the effort inaugurated by the Census Bureau. No intelligent action can be taken, nor success gained in any effort at sanitary reform looking to the prevention of disease, that has not the co-operation of the medical profession, and is based on a knowledge of the number of deaths and the causes that lead to them. A careful registration of all births, marriages and deaths, with all essential details of each, and a compilation therefrom under proper authority, is the basis, not only of sanitation and preventive medicine, but of social reforms, affecting health and longevity—such, for instance, as amending the marriage laws, and regulation of certain social evils at which we will only hint. Its importance can therefor not be overestimated.

It is hardly worth while, in talking to physicians, as we are now, to point out that only a knowledge of the number of deaths and the causes thereof will enable the authorities to remove those causes, so far as may be possible; and that a diminution in the number of deaths affects the average length of life of a people; and this, in turn affects nearly all economic conditions—adding thereby not only to the health and happiness of a people, but to the wealth, strength and prosperity of a nation.

IN REPLY to the letter from the Census Commissioner (published herewith), on the subject of a co-operative plan of mortality statistics, we stated that in Texas there is no State provision for registering, collecting and preserving the vital statistics of the people; or, as Dr. M. M. Smith, in his memorable address at the April meeting of the Texas State Medical Association at San Antonio, said: "Texas has a system of vital statistics for her pigs, but none for her people." We stated also that for twenty years or more the medical profession of Texas had vainly endeavored to impress upon governors and legislators the importance and necessity of a system of vital statistics, and that, hence, we believed that the co-operation asked for by the Census officials could not be expected of the Texas government unless required by an act of Congress.

It is true, in some towns and counties there is an alleged record kept of births and deaths, etc., and the (alleged) causes of the deaths, but there are no returns made to the State government, and no record of these things kept at the Capitol. There is no record at Austin, so far as I know, to show that any one was ever born in the State; that any one ever died in the State, or if one were born, there is nothing to show whether or not his parents were ever married; or of what color, nationality or "previous condition" his parents were. This is a disgrace to our boasted civilization.

In States where, as in Texas, there is no record kept by the State the Census Commissioner proposes as an alternative to send his enumerators to each town or at least to each county seat. Even there he will find that there is no record, or, if any, a very imperfect one kept.. Some towns keep a record, and some—the majority of them, in Texas—do not. Statistics gathered in this way will therefore be valueless; an omission of any feature of the returns, will, as Mr. Merriam points out, vitiate the whole. A partial return is worse than useless; it is misleading. I will give an illustration: The *Treasury* Department, through the *Marine Hospital Service* (why not the Agricultural ,or the War Department?) has issued a pamphlet entitled "*Mortality Statistics* in the *United States* for the year ending December 31, 1897 (from the annual report of *Marine Hospital Service*)." In this pamphlet we find—take Texas for our illustration—that *twenty-three* towns reported to the Marine Hospital Service—a total population of 214,000, with a total number of deaths 2486—an annual mortality of 11.60 per 1000 of the estimated population. Of these deaths 233 were ascribed to *phthisis pulmonalis.** ONE DEATH to smallpox! Forty-two to enteric fever. Measles, 2. Scarlet fever, 2. Diphtheria, 55. Whooping cough, 14. Now, as this pamphlet doubtless is accepted all over the world as an official report of the mortality in the States of the American Republic, it wil be seen how misleading it is. It would naturally create the impression that smallpox is exceedingly rare, in Texas for instance; whereas the "woods are full of it" nearly always, and the death rate at Laredo, where the disease became violently epidemic last fall and on which occasion the State of Texas removed patients to the pest house with the aid of a gatling gun and a troop of negro cavalry behind the health officers, was over twenty-six per cent. Hundreds, yea thousands, of cases of smallpox occur in Texas nearly every year.

Such publications as this alleged "Mortality Statistics of the United States" are a travesty, a *reductio ad absurdum*.

That the United States of America, in its second century of existence—"God's country"—the "great and glorious" Republic—one of the mighty nations of the world—should have no *system* of preserving the mortality statistics of its seventy odd millions of people is, indeed, most astounding. Congressmen are too busy making money to attend to little matters like *this*, tho'.

All of which emphasizes what the JOURNAL has been preaching for fifteen years "hand running": the necessity of a *Department of Public Health* in the U. S. government.

**AS TO SEWERS AND SEWAGE.**

THAT THE "great and glorious" has no system of vital and mortuary statistics is not the only thing in connection with the shameful neglect of the public health and prosperity that is astounding. That the United States has no law to protect its rivers and streams from pollution, for instance, is astounding. Chicago is about to

---

*The report of the health officer of San Antonio, Texas, shows (see TEXAS MEDICAL JOURNAL, August, 1899, p. 107) that of 1440 deaths from all causes in that city for the year ending June 30, 1899, 315 were from consumption, San Antonio is included in the Marine Hospital report cited; though Galveston, Austin, Dallas, Fort Worth and Waco—five of the largest cities—are not.—ED.

convert the Mississippi river into one grand sewer into which to dump the millions of tons of human excrement of her three millions of people, and all other filth incident to a swarming population. Congressmen are too busy making money to attend to a little matter like this, also. New York gets her drinking water from the Croton river, and mounted policemen see that not a pig pen, nor a cow lot, nor a privy is so located, within the area of the entire water-shed of the Croton, that its filth is washed into the basin. At Austin last spring our $2,000,000 reservoir was the receptacle of the sewage from a camp of five thousand troops and one thousand horses—a disgraceful fact which we have mentioned before. The Chicago people will utilize the great river as a sewer with as little regard, seemingly, for the poor devils who live lower down—say as St. Louis, Cairo, Memphis, Vicksburg, New Orleans, etc., and the millions of people along the banks who drink the water—as had Old Augeus for those cities and peoples along the Pineus and the Alpheus when Hercules turned those streams through the old man's horse lot. Another such disregard of all sanitary science,—disregard of the lessons of history, disregard for the welfare of the people, has not occurred since those days.

Will not the people of St. Louis attempt to prevent it?

HERE'S another, from an unexpected source. Read it:

"Defective plumbing and had sewerage are two common enemies of all large cities, and Louisville is no exception to the rule. Within the eastern limits of the city is a filthy, sluggish stream into which all kinds of sewage is constantly pouring. The emanations from this cesspool are a menance to the health of all who live adjacent to it, and it is undoubtedly the cause of much sickness. It would cost only a small amount to grade this stream so as to allow this filth to find its way *directly into the river without delay.** It is the accumulation of the filth in the low places and sluggish parts of this stream that makes it so obnoxious."—*Amer. Prac. and News, Louisville, Ky.*

We'll get the nuisance off of our premises by unloading it on the other fellows.

ASIDE from sanitary considerations sewage has a great economic value, and should be utilized. Tons of animal matter, such as constitute the sewage of a large city, can be and should be utilized as fertilizers, as is done in Paris today. A writer in *The Humanitarian* goes so far as to propose that the bodies of all paupers be turned

*Italics ours.—ED.

over to a firm to be converted into fertilizers to grow corn and potatoes for the living; not a bad idea.

*Talk about the Filipinos! Are we* a civilized nation? We appear to be totally unenlightened on sanitary science at least: I mean—Congressmen and legislators—and "governors."

All of which goes to show the need of a Public Health Department of the U. S. government. But Congressmen are too busy making money to attend to a little matter like *this*, also.

